# Long noncoding RNA HULC accelerates the growth of human liver cancer stem cells by upregulating CyclinD1 through miR675-PKM2 pathway via autophagy

**DOI:** 10.1186/s13287-019-1528-y

**Published:** 2020-01-03

**Authors:** Chen Wang, Xiaoxue Jiang, Xiaonan Li, Shuting Song, Qiuyu Meng, Liyan Wang, Yanan Lu, Xiaoru Xin, Hu Pu, Xin Gui, Tianming Li, Dongdong Lu

**Affiliations:** Shanghai Putuo District People’s Hospital, School of Life Science and Technology, Tongji University, Shanghai, 200092 China

**Keywords:** HULC, CyclinD1, miR675, PKM2, Autophagy

## Abstract

**Background:**

The functions of HULC have been demonstrated in several cancers. However, its mechanism has not been elucidated in human liver cancer stem cells.

**Methods:**

Liver cancer stem cells were isolated from Huh7 cells; gene infection and tumorigenesis test in vitro and in vivo were performed.

**Results:**

We demonstrate that HULC promotes growth of liver cancer stem cells in vitro and in vivo*.* Mechanistically, HULC enhances the expression of Sirt1 dependent on miR675 and then induces the cellular autophagy through Sirt1. HULC enhances CyclinD1 and thereby increases pRB and inhibited P21 WAF1/CIP 1 via autophagy-miR675-PKM2 pathway in human liver cancer stem cells. Ultimately, our results demonstrate that CyclinD1 is required for the oncogenic functions of HULC in liver cancer stem cells.

**Conclusions:**

It reveals the key molecular signaling pathways for HULC and provides important basic information for finding effective tumor therapeutic targets based on HULC.

## Introduction

HULC has been studied in several cancers and promotes tumorigenesis [1–4]. Furthermore, HULC polymorphisms are associated with hepatocellular cancer risk and prognosis [5, 6]. In addition, HULC enhances autophagy [7] and facilitates hepatocellular carcinoma genesis [8]. Furthermore, HULC regulates the expression of bone morphogenetic protein9 (*BMP9)* [9]. Interestingly, HULC acts as an oncogene [10] and inhibits apoptosis [11] and promotes invasion [12, 13]. Furthermore, HULC stabilizes Sirt1 and decreases the chemosensitivity [14]. Moreover, HULC aggravates the cellular proliferation by regulating telomere repeat-binding factor2 [15] and CUDR, β-Catenin [16], and IGF2 mRNA-binding protein 1 (IGF2BP1) [17].

In this study, HULC is associated with miRNA675, Sirt1, CyclinD1, and autophagy. A study indicates that miR-675 enhances cell proliferation [18, 19] and Smads/miR-675/TGFβR1 axis modulates the proliferation [20]. Moreover, sPIF promotes myoblast differentiation via the H19/miR-675/let-7 pathways [21] Furthermore, miR-675 mediates therapeutic effect [22]. A study indicates that SIRT1 is implicated in stem cell homeostasis. In particular, Conditional *Sirt1* deletion in the hematopoietic stem and progenitor system promotes hematopoietic stem and progenitor cell (HSPC) expansion under stress conditions [23]. Moreover, SIRT1 enhances progression and epithelial-mesenchymal transition in several cancer [24, 25]. Furthermore, CyclinD1 promotes the cancer cell growth dependent on autophagy [26]. A study shows that CyclinD1 complement p16 acts as tumor marker [27] and shows heterogeneous expression of pRb and CyclinD1 [28]. Importantly, autophagy is essential in cellular processes [29]. For example, downregulation of CD44v6 inhibits autophagy in colorectal cancer HT29 cells [30], and LncRNA CCAT1 functions as apoptosis inhibitor via autophagy inhibition [31] and upregulated lysine-specific demethylase 4B by autophagy [32]. Notably, BCR signaling contributes to autophagy regulation [33].

In this study, our observations suggest that HULC promotes progression of liver cancer stem cells dependent on CyclinD1. It provides important basic information for finding effective tumor therapeutic targets.

## Materials and methods

### Cell infection and transfection

Cells were infected with lentivirus and transfected with DNA plasmids according to the manufacturer’s instructions (also see Additional file 1).

### MicroRNA detection

Real-time RT-PCR-based detection of mature miR-675 was achieved with the miRNA Detection kit and miR-675-specific upstream primers (5′-TGGTGCGGAGAGGGCCCACAGTG-3′).

### RNA immunoprecipitation (RIP)

Ribonucleoprotein particle-enriched lysates were incubated with protein A/G-plus agarose beads (Santa Cruz, Biotechnology, Inc.CA) together with the primary antibody or normal IgG for 4 h at 4 °C. Beads were subsequently washed and RNAs were then isolated. RT-PCR was performed according to the manufacturer’s instructions.

### Cells proliferation CCK8 assay

Cells were grown in complete medium for CCK8 assay according to the manufacturer’s instructions. Cell growth curve was based on the values of OD450.

### Colony-formation efficiency assay

Cell colonies on the dish were stained with Crystal Violet (Henan Tianfu Chemical Co., Ltd.), and the colonies were counted according to the manufacturer’s instructions.

### Xenograft transplantation in vivo

Four-week male athymic Balb/C mice were purchased from Shi Laike Company (Shanghai, China). The athymic Balb/C mice were injected at the armpit area subcutaneously with suspension of cells. The wet weight of each xenograft was determined for each mouse. The use of mice for this work was reviewed and approved by the institutional animal care and use committee in accordance with China National Institutes of Health guidelines.

## Results

### HULC promotes growth of liver cancer stem cells

**Fig. 1:**
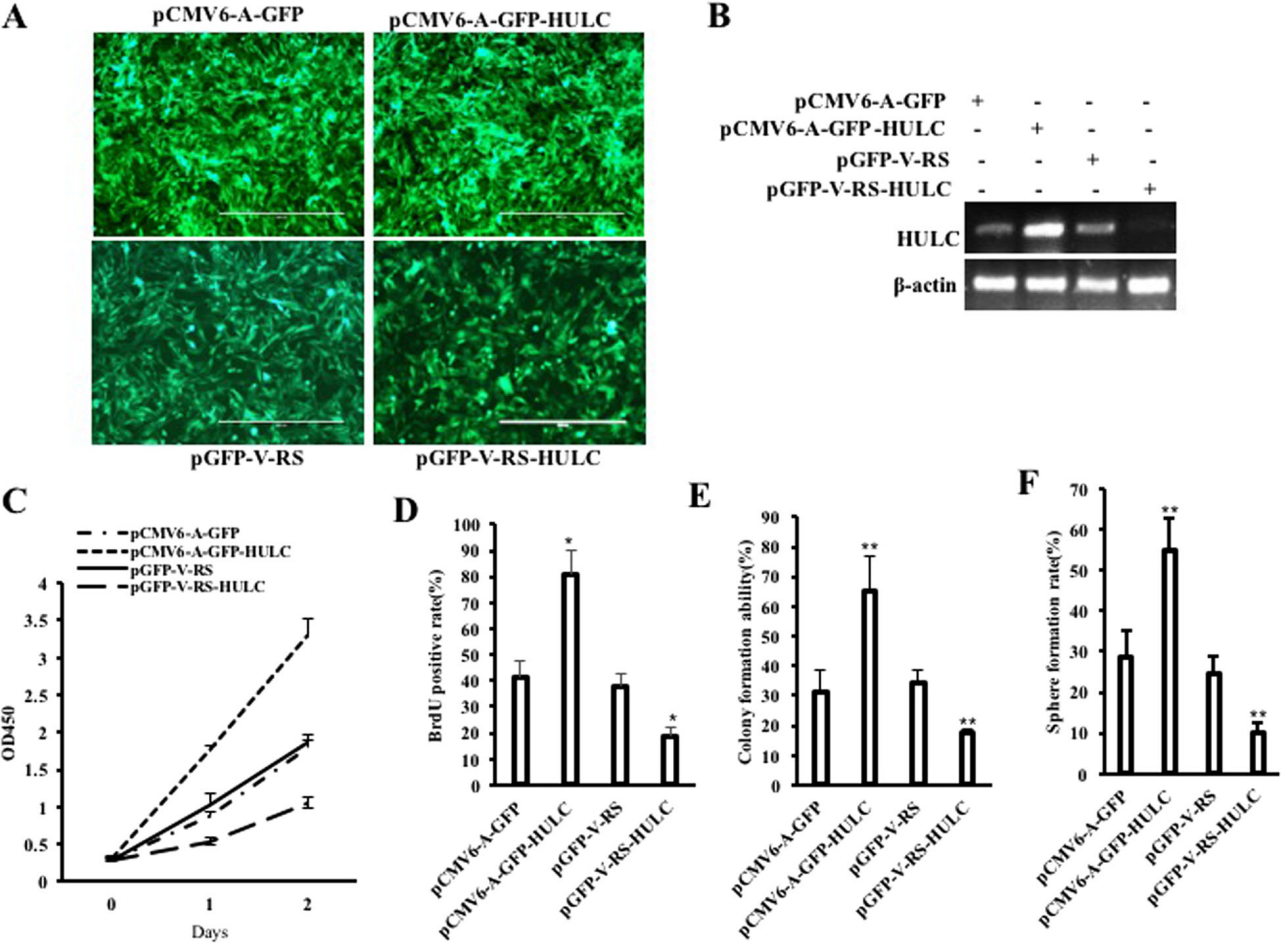
HULC accelerates hLCSCs growth in vitro*.* **a** the photography of transfected cells. **b** RT-PCR analysis of expression of HULC in hLCSCs. β-actin was used as internal control. **c** Cell growth assay using CCK8. **d** S phase cells assay using BrdU. **e** Soft-agar colony formation assay. **f** Cell sphere formation ability

To demonstrate the effect of HULC on human liver cancer stem cells, we perform the tumorigenesis test in vitro. First, human liver cancer stem cells were isolated from Huh7 cells. Cells that meet the four indexes of CD133+, CD44+, CD24+, and EpCAM+ are defined as human liver cancer stem cells (hLCSCs), and cells that satisfy the four indexes of CD133-, CD44-, CD24-, and EpCAM are defined as non-hepatoma stem cells (non-hLCSCs). CD44, CD24, and EpCAM are expressed in hLCSCs, but not in non-hLCSCs (Additional file 1: Figure S1). Moreover, the sphere formation rate is 0.153 ± 0.0372% in the hLCSCs group, and the sphere formation rate is 0 in the non-hLCSCs group (0.153 ± 0.0372% vs 0, *P* = 0.00079 < 0.01) (Additional file 1: Figure S2A). The weight of xenograft tumors is 0.68 ± 0.19 g in the hLCSCs group, and the weight of xenograft tumors is 0 g in the non-hLCSCs group (0.68 ± 0.19 g vs 0, *P* = 0.00000098 < 0.01) (Additional file 1: Figure S2B). Next, we established four stable hLCSC lines transfected with pCMV6-A-GFP (GFP ctrl group), pCMV6-A-GFP-HULC (HULC group), pGFP-V-RS (RNAi ctrl group), and pGFP-V-RS-HULC (HULCi group), respectively (Fig. 1a). As shown in Fig. 1b, HULC expression was significantly enhanced in the HULC group compared with the GFP ctrl group and reduced in the HULCi group compared with the RNAi ctrl group. As shown in Fig. 1c, the growth ability was significantly increased in the HULC group compared to the GFP ctrl group (*P* < 0.01) and decreased in the HULCi group compared to the RNAi ctrl group compared to the GFP ctrl group (*P* < 0.01). Moreover, the proportion of BrdU-positive cells in the HULC group was significantly increased in the HULC group compared to the GFP ctrl group (*P* < 0.01) and decreased in the HULCi group compared to the RNAi ctrl group compared to GFP the ctrl group (*P* < 0.01) (Fig. 1d). Furthermore, the soft-agar colony formation rate was significantly increased in the HULC group compared to the GFP ctrl group (31.09 ± 7.29% vs 65.30 ± 11.58%, *P* = 0.0053 < 0.01) and decreased in the HULCi group compared with the RNAi ctrl group (34.049 ± 4.79% vs 17.34 ± 1.37%, *P* = 0.0102 < 0.05) (Fig. 1e). The sphere-formation rate of hLCSCs was significantly increased in the HULC group compared to the GFP ctrl group (28.74 ± 6.47% vs 54.71 ± 8.19%, *P* = 0.0008 < 0.01) and decreased in the HULCi group compared with the RNAi ctrl group (24.52 ± 4.31 vs 10.03 ± 2.67%, *P* = 0.031 < 0.05) (Fig. 1f). Collectively, these results suggest that HULC promotes the growth in vitro of liver cancer stem cells.

### HULC accelerates growth of hLCSCs in vivo

**Fig. 2:**
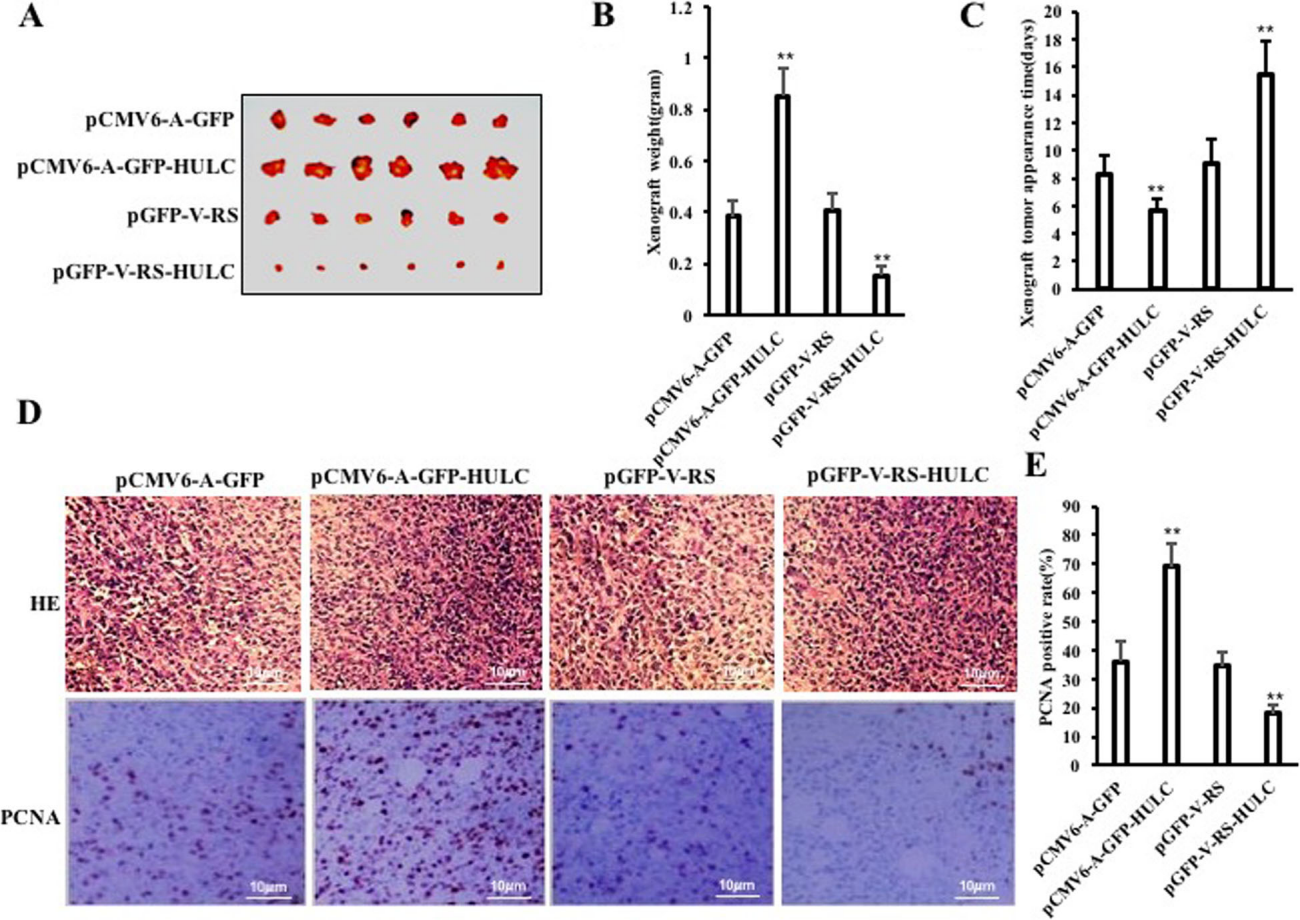
HULC accelerates hLCSCs growth in vivo*.* **a** The photograph of xenograft tumors derived from four hLCSC lines injected into mice. **b** The wet weight of xenograft tumors. **c** The appearance time of xenograft tumors. **d** Histological hematoxylin-eosin (HE) staining (upper pictures) and anti-PCNA immunostaining (lower pictures) of xenograft tumors (original magnification × 100). **e** PCNA-positive cell analysis of xenograft tumors

To investigate the effect of HULC on hLCSCs in vivo, the four stable hLCSC lines were injected subcutaneously into Balb/C mice, respectively. As shown in Fig. 2a and b, compared with the GFP ctrl group, the weight of xenograft tumor was increased approximately by twofold in the HULC group (0.385 ± 0.057 g vs 0.852 ± 0.108 g, *p* = 0.000007933 < 0.01); however, compared with the RNAi ctrl group, the weight of xenograft tumor was decreased approximately by one third in the HULCi group (0.45 ± 0.068 g vs 0.153 ± 0.0372 g, *p* = 0.000224907 < 0.01). Furthermore, the xenograft tumors appeared earlier in the HULC group than in the GFP ctrl group (8.33 ± 1.37 days vs 5.67 ± 0.816 days, *p* = 0.0014788 < 0.01), whereas those appeared later in the HULCi group than in the RNAi ctrl group (9.0 ± 1.79 days vs 15.5 ± 2.43 days, *p* = 0.000184 < 0.01) (Fig. 2c). Furthermore, xenograft tumor differentiation was poorer in the HULC group than in the GFP ctrl group, whereas xenograft tumor differentiation was well in the HULCi group than in the RNAi ctrl group (Fig. 2d). Furthermore, the PCNA-positive rate was significantly higher in the HULC group than in the GFP ctrl group (36.15 ± 7.25% vs 69.99 ± 8.24%, *p* = 0.00041 < 0.01) and lower in the HULCi group than in the RNAi ctrl group (34.62 ± 4.94% vs 18.19 ± 2.67%, *p* = 0.00029 < 0.01) (Fig. 2d, e). Together, these results suggest that HULC accelerates growth of liver cancer stem cells in vivo.

### HULC increases the miR675 in liver cancer stem cells

**Fig. 3:**
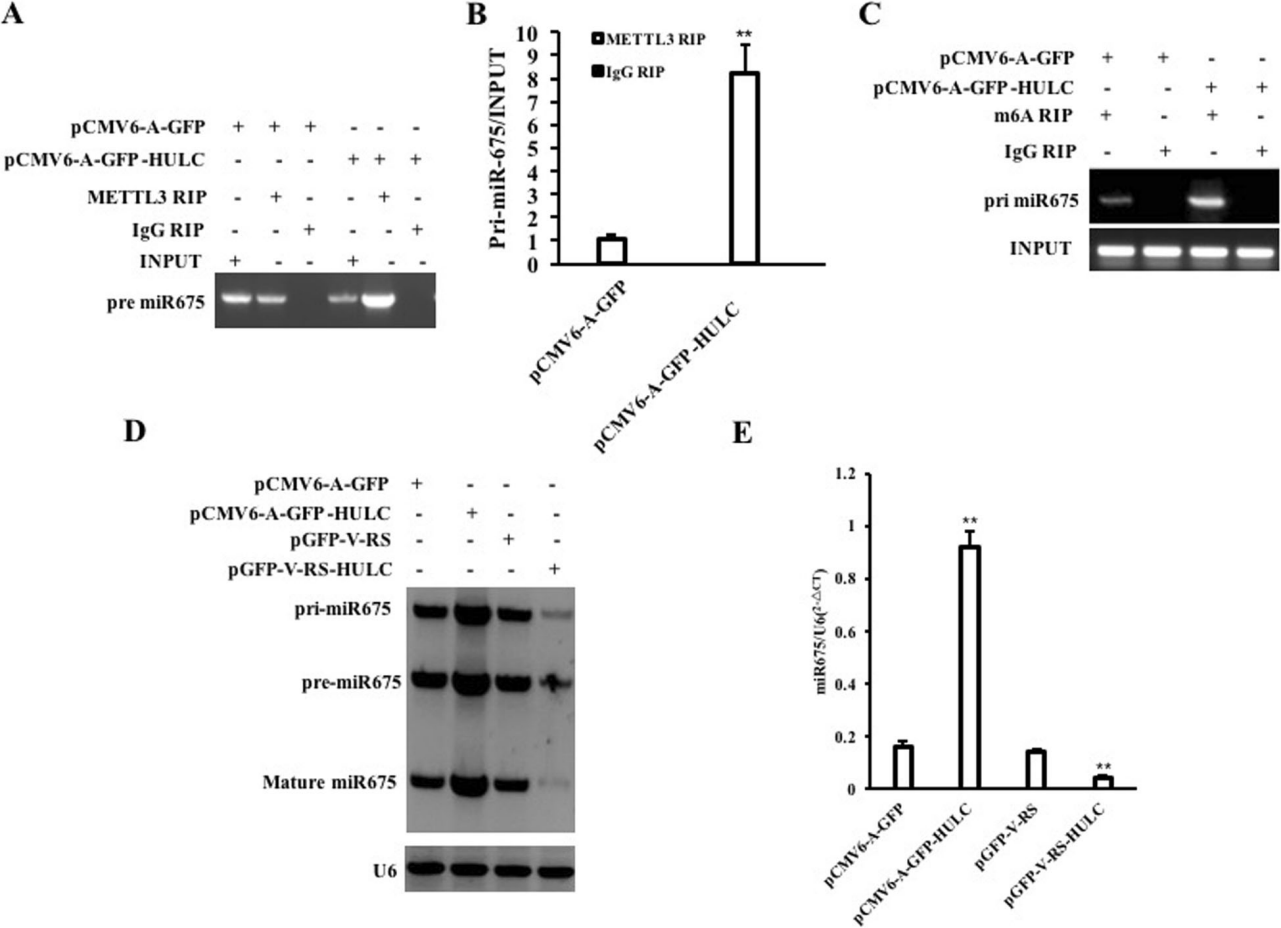
HULC increases miR675. **a** RNA immunoprecipitation (RIP) with anti-METTL3 followed by RT-PCR. IgG RIP served as the negative control. **b** Real-time RNA Immunoprecipitation (RIP) with anti-METTL3 followed by RT-PCR. IgG RIP served as the negative control. **c** RNA Immunoprecipitation (RIP) with anti-m6A followed by RT-PCR with Pri-miR675 promoter primers. IgG RIP served as the negative control. **d** Northern blotting with Biotin-miR675 probe. U6 served as the internal control. **e** Real-time RT-PCR with miR675 primers. U6 served as an internal control

Given that HULC promotes the growth of liver cancer stem cells and miR675 is associated with oncogenesis, we consider whether HULC regulates the expression of miR675. To address this hypothesis, we measured the level of RNA methylation of pri-miR675 in hLCSCs. Our results showed that excessive HULC increases and HULC knockdown decreased the binding of METTL3 (a RNA methyltransferase) to pri-miR675 compared to the control group (Fig. 3a). Furthermore, real-time RIP results showed that the binding of METTL3 (a RNA methyltransferase) to pri-miR675 was increased in the pCMV6-A-GFP-HULC group compared to the pCMV6-A-GFP group and decreased in the pGFP-V-RS-HULC group compared to the pGFP-V-RS group (Fig. 3b). In particular, pri-miR675, pre-miR675, and mature miR-R675 were significantly increased in the pCMV6-A-GFP-HULC group compared to the pCMV6-A-GFP group and decreased in the pGFP-V-RS-HULC group compared to the pGFP-V-RS group (Fig. 3c). Furthermore, mature miR675 was increased in the pCMV6-A-GFP-HULC group compared to the pCMV6-A-GFP group and decreased in the pGFP-V-RS-HULC group compared to the pGFP-V-RS group (Fig. 3d). Although pre-miR675, pre-miR675, and mature mi-R675 were significantly increased in the pCMV6-A-GFP-HULC group compared to the pCMV6-A-GFP group, it was significantly not altered in the pCMV6-A-GFP-HULC+pGFP-V-RS-METTL3 group compared to the pCMV6-A-GFP group (Additional file 1: Figure S3A&B). Collectively, these observations suggest that HULC enhances the expression and maturity of miR675 dependent on METTL3.

### HULC promotes the expression of Sirt1 dependent on miR675

**Fig. 4:**
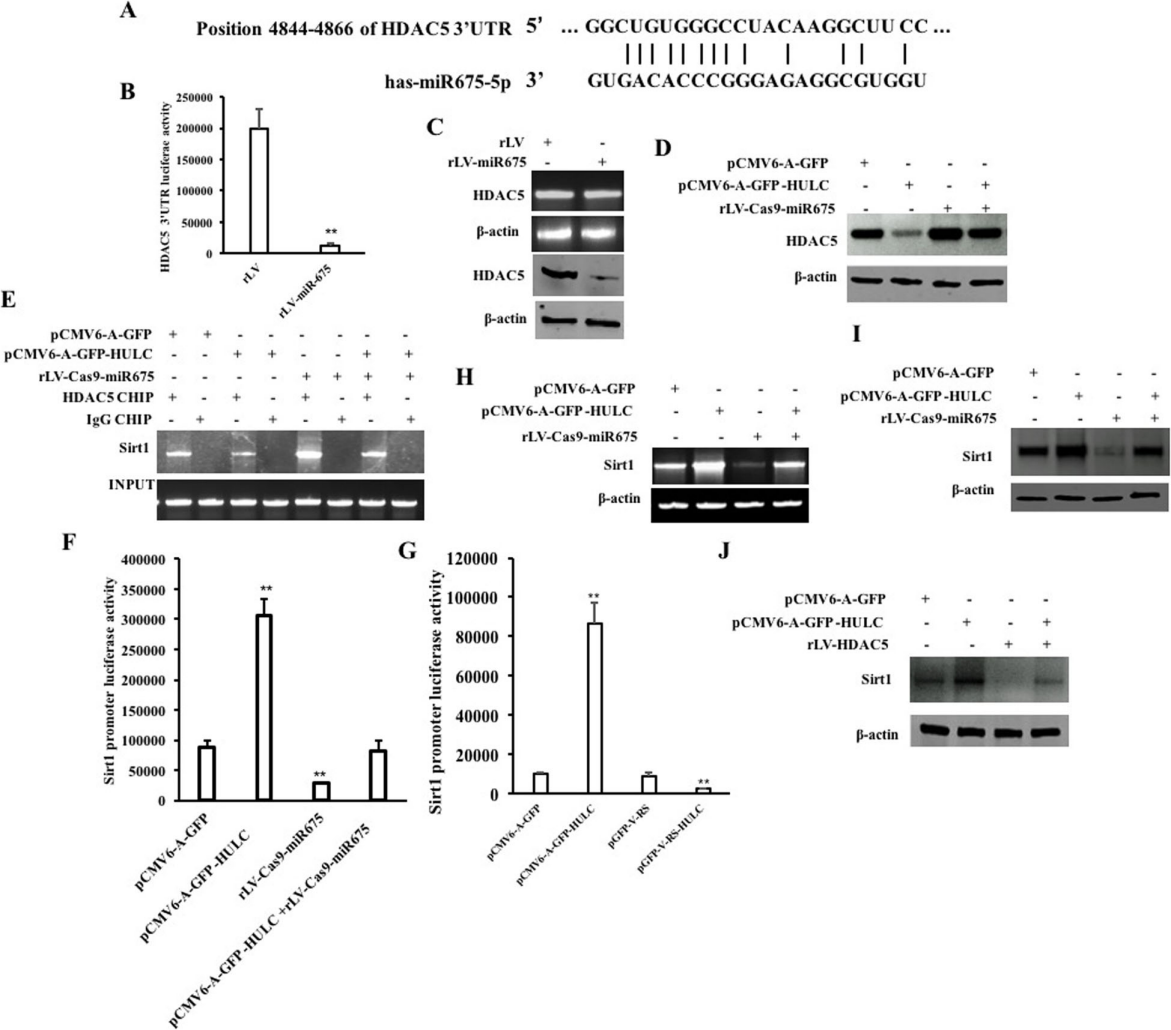
HULC enhances the interaction between LC3 and Sirt1. **a** The bioinformatics analysis of miR675 targeting for HDAC5. **b** The analysis of HDAC5 3′UTR luciferase reporter activity. **c** RT-PCR analysis for HDAC5 and Western blotting with anti-HDAC5. **d** Western blotting with anti-HDAC5. β-actin was used as internal control. **e** CHIP assay with anti-HDAC5 followed by PCR with Sirt1 promoter primers. **f** The analysis of Sirt1 promoter luciferase reporter activity. **g** The analysis of Sirt1 promoter luciferase reporter activity. **h** RT-PCR with Sirt1 cDNA primers. β-actin was used as internal control. **i** Western blotting with anti-Sirt1. β-actin was used as internal control. **j** Western blotting with anti-Sirt1 in the pCMV6-A-GFP group, pCMV6-A-GFP-HULC group, and pCMV6-A-GFP-HULC+rLV-HDAC5 group. β-actin was used as internal control

Given that HULC increases the expression of miR675, we will try to consider whether HULC regulates expression of Sirt1 via miR675. As shown in Fig. 4a, mature miR675 matches 3′ untranslational region (UTR) on histone deacetylase 5(HDAC5) mRNA via eight-seed sequence. Next, as shown in Fig. 4b, although the DHAC5 3′UTR luciferase activity was significantly reduced in the rLV-miR675 group compared to the rLV control group (199,362.03 ± 32,442.268 vs 12,057.69 ± 4192.57, *p* = 0.00375 < 0.01), it was significantly not altered in the rLV-miR675 group compared to the rLV group (118,226.40 ± 14,210.88 vs 105,230.04 ± 22,650.11, *p* = 0.22704 > 0.05) (Additional file 1: Figure S4). Although the DHAC5 mRNA was not significantly altered between the rLV-miR675 group and rLV control group, the expression of DHAC5 was significantly reduced in the rLV-miR675 group compared to the rLV control group (Fig. 4c). Although the expression of DHAC5 was significantly reduced in the pCMV6-A-GFP-HULC group and increased in the rLV-Cas9-miR675 group compared to the pCMV6-A-GFP group, it was significantly not altered in the pCMV6-A-GFP-HULC plus rLV-Cas9-miR675 group compared to the pCMV6-A-GFP group (Fig. 4d). In particular, the loading of DHAC5 on the Sirt1 promoter region was significantly reduced in the pCMV6-A-GFP-HULC group and increased in the rLV-Cas9-miR675 group compared to the pCMV6-A-GFP group. However, the expression of DHAC5 was significantly not altered in the pCMV6-A-GFP-HULC plus rLV-Cas9-miR675 group compared to the pCMV6-A-GFP group (Fig. 4e). Furthermore, although the luciferase activity of Sirt1 promoter was significantly increased in the pCMV6-A-GFP-HULC group (87,825.04 ± 10,954.98 vs 306,040.71 ± 27,824.28, *p* = 0.0042 < 0.01) and reduced in the rLV-Cas9-miR675 group compared to the pGFP-V-RS group compared to the pCMV6-A-GFP group (87,825.04 ± 10,954.98 vs 28,809.006 ± 999.09, *p* = 0.0053 < 0.01), it was significantly not altered in the pCMV6-A-GFP-HULC plus rLV-Cas9-miR675 group compared to the pCMV6-A-GFP group (87,825.04 ± 10,954.98 vs 82,539.303 ± 16,170.81, *p* = 0.3625 > 0.05) (Fig. 4f). Moreover, the luciferase activity of Sirt1 promoter was significantly increased in the pCMV6-A-GFP-HULC group compared to the pCMV6-A-GFP group (10,032.48 ± 1131.55 vs 86,393.99 ± 10,824.39, *p* = 0.003835 < 0.01) and reduced in the pGFP-V-RS-HULC group compared to the pGFP-V-RS group (9143.86 ± 1613.94 vs 2522.18 ± 429.04, *p* = 0.0059436 < 0.01) (Fig. 4g). Ultimately, the expression of Sirt1 was significantly increased in the pCMV6-A-GFP-HULC group and decreased in the rLV-Cas9-miR675 group compared to the pCMV6-A-GFP group. However, the expression of Sirt1 was significantly not altered in the pCMV6-A-GFP-HULC plus rLV-Cas9-miR675 group compared to the pCMV6-A-GFP group (Fig. 4h, i). Moreover, although the expression of Sirt1 was significantly increased in the pCMV6-A-GFP-HULC group and decreased in the rLV-Cas9-miR675 group compared to the pCMV6-A-GFP group, it was significantly not altered in the pCMV6-A-GFP-HULC plus rLV-HDAC5 group compared to the pCMV6-A-GFP group (Fig. 4j). Collectively, these observations indicate that HULC enhances the expression of Sirt1 dependent on miR675-HDAC5.

### HULC increases the autophagy through Sirt1

**Fig. 5:**
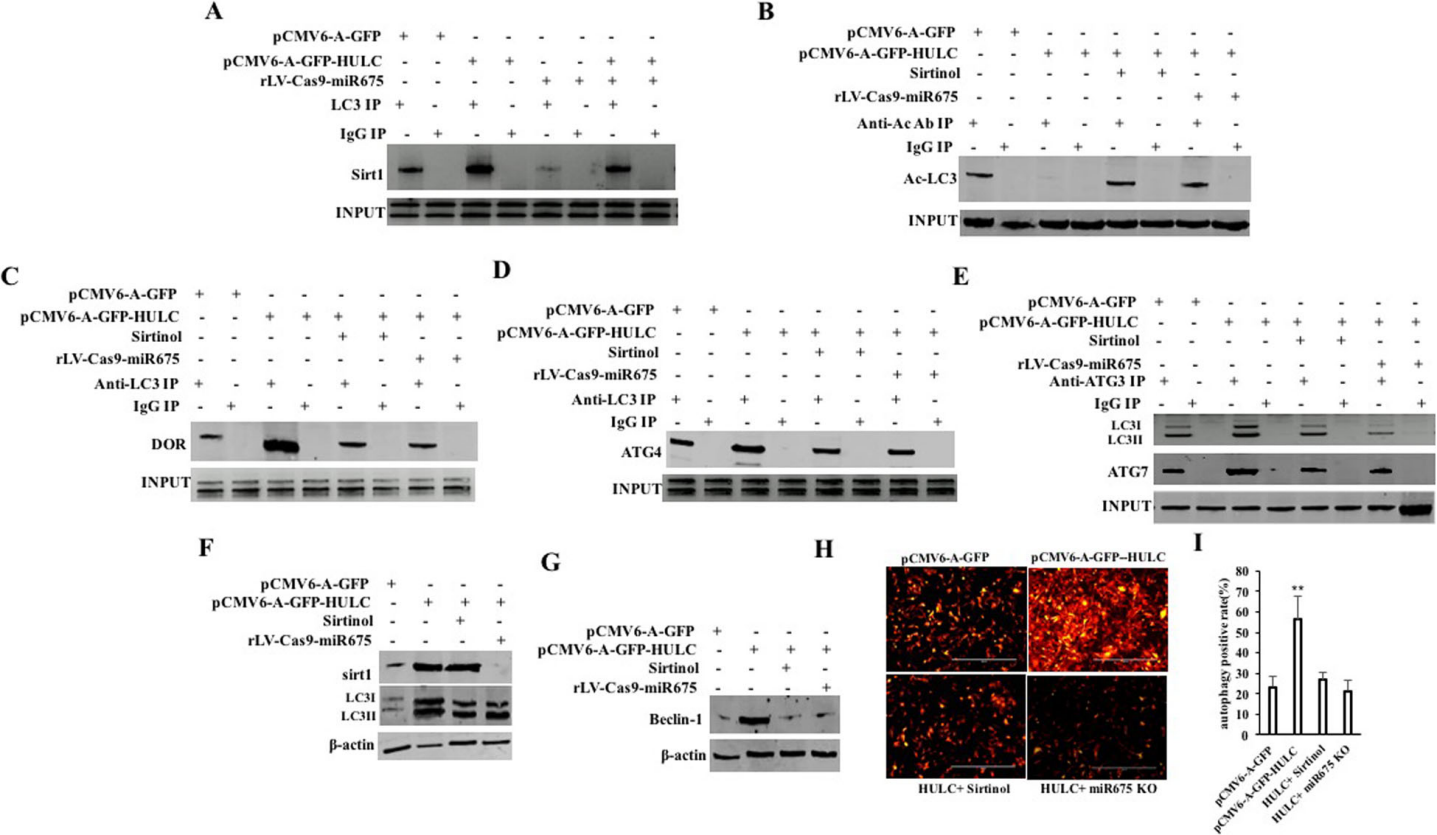
HULC triggers the autophagy through Sirt1. **a** Co-IP with anti-LC3 followed by Western blotting with anti-Sirt1. **b** Co-IP with anti-Ac followed by Western blotting with anti-LC3. **c** Co-IP with anti-LC3 followed by Western blotting with anti-DOR. **d** Co-IP with anti-LC3 followed by Western blotting with anti-ATG4. **e** Co-IP with anti-ATG3 followed by Western blotting with anti-LC3 and ATG7. IgG IP served as negative control. **f** Western blotting with anti-Sirt1 and anti-LC3, respectively. **g** Western blotting with anti-Beclin-1 in four hLCSC lines. **h** The observation for autophagy (LC3-RFP) in hLCSCs. **i** The autophagy rate

Given that HULC increases the expression of Sirt1 which is associated with deacetylation of LC3, we consider whether HULC influences on the autophagy through Sirt1 in liver cancer stem cells. First, although the interaction between Sirt1 and LC3 was significantly increased in the pCMV6-A-GFP-HULC group and decreased in the rLV-Cas9-miR675 group compared to the pCMV6-A-GFP group, it was significantly not altered in the pCMV6-A-GFP-HULC plus rLV-Cas9-miR675 group compared to the pCMV6-A-GFP group (Fig. 5a). As shown in Fig. 5b, the Ac-LC3 was significantly decreased in the pCMV6-A-GFP-HULC group compared to the pCMV6-A-GFP group. However, the Ac-LC3 was not significantly altered in the pCMV6-A-GFP-HULC plus Sirtinol (a Sirt1 inhibitor) group and the pCMV6-A-GFP-HULC plus rLV-Cas9-miR675 group compared to pCMV6-A-GFP group, respectively. Therefore, although the interaction between LC3 and DOR was significantly increased in the pCMV6-A-GFP-HULC group compared to the pCMV6-A-GFP group, it was not significantly altered in the pCMV6-A-GFP-HULC plus Sirtinol group and the pCMV6-A-GFP-HULC plus rLV-Cas9-miR675 group compared to the pCMV6-A-GFP group (Fig. 5c). Strikingly, the interaction between LC3 and ATG4 was significantly increased in the pCMV6-A-GFP-HULC group compared to the pCMV6-A-GFP group. However, the interaction between LC3 and DOR was not significantly altered in the pCMV6-A-GFP-HULC plus Sirtinol group and the pCMV6-A-GFP-HULC plus rLV-Cas9-miR675 group compared to the pCMV6-A-GFP group, respectively (Fig. 5d). Furthermore, the interaction between LC3 and ATG3 was significantly increased in the pCMV6-A-GFP-HULC group compared to the pCMV6-A-GFP group. However, the interaction between LC3 and ATG3 was not significantly altered in the pCMV6-A-GFP-HULC plus Sirtinol group and the pCMV6-A-GFP-HULC plus rLV-Cas9-miR675 group and compared to the pCMV6-A-GFP group, respectively (Fig. 5e). And, the interaction between ATG3 and ATG7 was significantly increased in the pCMV6-A-GFP-HULC group compared to the pCMV6-A-GFP group. However, the interaction between ATG3 and ATG7 was not significantly altered in the pCMV6-A-GFP-HULC plus Sirtinol group and the pCMV6-A-GFP-HULC plus rLV-Cas9-miR675 group compared to the pCMV6-A-GFP group, respectively (Fig. 5e). Thus, the activated LC3II was significantly enhanced in the pCMV6-A-GFP-HULC group compared to the pCMV6-A-GFP group. However, the activated LC3II was not significantly altered in the pCMV6-A-GFP-HULC plus Sirtinol group and the pCMV6-A-GFP-HULC plus rLV-Cas9-miR675 group compared to the pCMV6-A-GFP group, respectively (Fig. 5f). In particular, the beclin1 was significantly increased in the pCMV6-A-GFP-HULC group compared to the pCMV6-A-GFP group. However, the beclin1 was not significantly altered in the pCMV6-A-GFP-HULC plus Sirtinol group and the pCMV6-A-GFP-HULC plus rLV-Cas9-miR675 group compared to the pCMV6-A-GFP group, respectively (Fig. 5g). Ultimately, the autophagy was significantly enhanced in the pCMV6-A-GFP-HULC group compared to the pCMV6-A-GFP group (23.38 ± 5.27% vs 56.41 ± 11.38%, *p* = 0.00902 < 0.01). However, the autophagy was not significantly altered in the pCMV6-A-GFP-HULC plus Sirtinol group and the pCMV6-A-GFP-HULC plus rLV-Cas9-miR675 group compared to the pCMV6-A-GFP group, respectively (23.38 ± 5.27% vs 26.93 ± 3.56%, *p* = 0.10548 > 0.05; 23.38 ± 5.27% vs 21.38 ± 5.41%, *p* = 0.361978 > 0.05) (Fig. 5h, i). Collectively, these observations suggest that HULC increases the autophagy dependent on Sirt1.

### HULC enhances CyclinD1 to increase pRB and inhibit P21 WAF1/CIP 1 via autophagy-PKM2 pathway

**Fig. 6:**
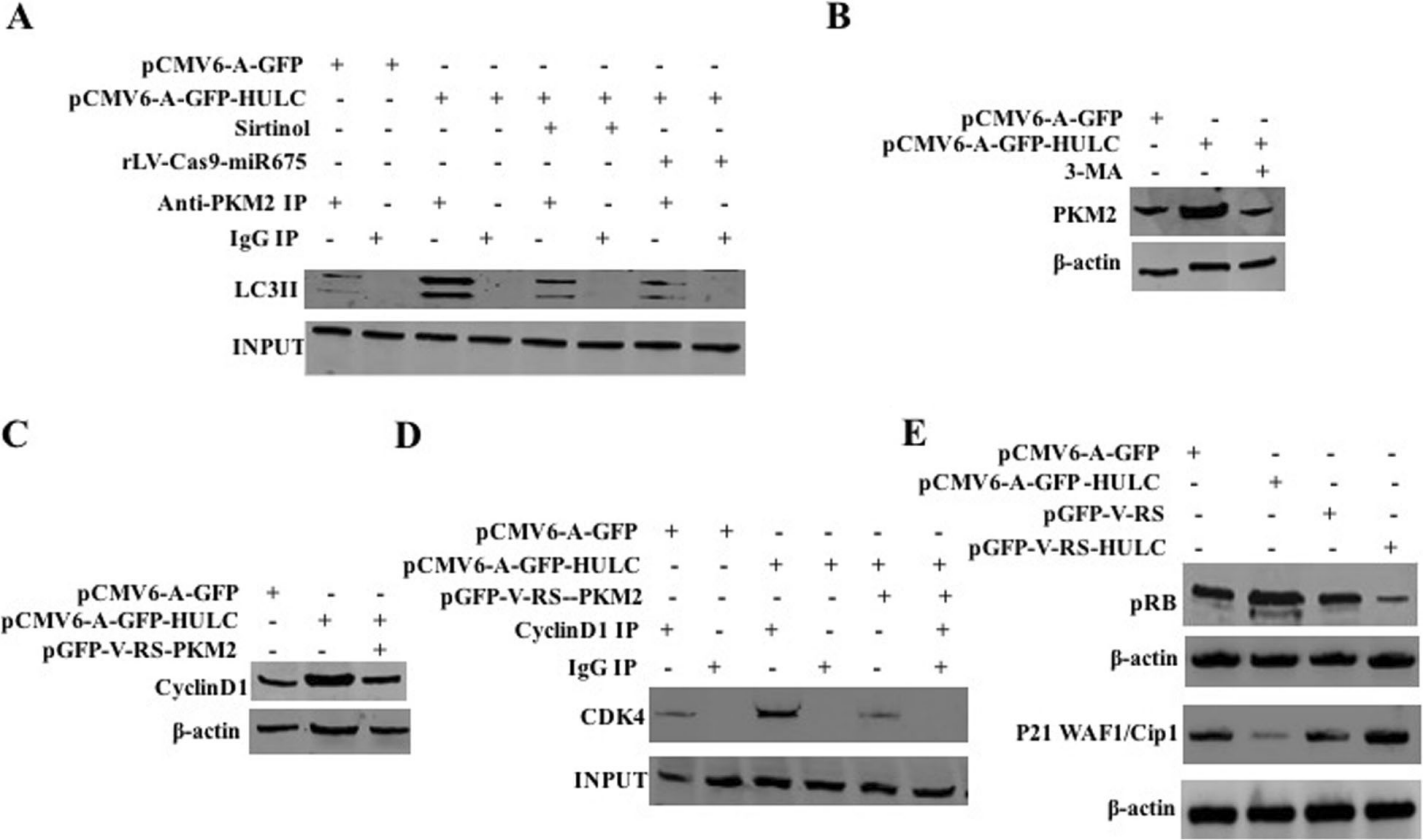
HULC enhances pRB and inhibit P21 WAF1/CIP1 in live cancer stem cells. **a** Co-IP with anti-PKM2 followed by Western blotting with anti-LC3. **b** Western blotting with anti-PKM2 in three hLCSC lines. **c** Western blotting with anti-CyclinD1 in three hLCSC lines. **d** Co-IP with anti-CyclinD1 followed by Western blotting with anti-CDK4. IgG IP served as negative control. Western blotting with anti-CDK4 served as INPUT. **e** Western blotting with anti-pRB and anti-P21WAF1/Cip1. β-actin served as internal control

To address whether HULC influences on the PKM2 in liver cancer stem cells by autophagy, we first analyze the interaction between LC3II and Pyruvate Kinase M2 (PKM2) in liver cancer stem cells. As shown in Fig. 6a, the interaction between LC3II and PKM2 was significantly enhanced in the pCMV6-A-GFP-HULC group compared to the pCMV6-A-GFP group. However, the interaction between LC3II and PKM2 was not significantly altered in the pCMV6-A-GFP-HULC plus Sirtinol group and the pCMV6-A-GFP-HULC plus rLV-Cas9-miR675 group compared to the pCMV6-A-GFP group, respectively. Moreover, the expression of PI3K and PKM2 was significantly increased in the pCMV6-A-GFP-HULC group compared to the pCMV6-A-GFP group. However, the expression of PI3K and PKM2 was not significantly altered in the pCMV6-A-GFP-HULC plus 3-methyladenine (*3-MA*) group compared to the pCMV6-A-GFP group, respectively (Fig. 6b). Therefore, the expression of CyclinD1 was significantly increased in the pCMV6-A-GFP-HULC group compared to the pCMV6-A-GFP group. However, the expression of CyclnD1 was not significantly altered in the pCMV6-A-GFP-HULC plus rLV-Cas9-PKM2 group compared to the pCMV6-A-GFP group, respectively (Fig. 6c). Moreover, the interaction between CDK4 and CyclinD1 was significantly enhanced in the pCMV6-A-GFP-HULC group compared to the pCMV6-A-GFP group. However, the interaction between CDK4 and CyclinD1 was significantly not altered in the pCMV6-A-GFP-HULC plus pGFP-V-RS-PKM2 group compared to the pCMV6-A-GFP group (Fig. 6d). Finally, pRB was significantly increased in the pCMV6-A-GFP-HULC group compared to the pCMV6-A-GFP group and reduced in the pGFP-V-RS-HULC group compared to the pGFP-V-RS group, and P21WAF1/Cip1 was significantly decreased in the pCMV6-A-GFP-HULC group compared to the pCMV6-A-GFP group and increased in the pGFP-V-RS-HULC group compared to the pGFP-V-RS group (Fig. 6e). Collectively, these observations suggest that HULC enhances CyclinD1 to increase pRB and inhibit P21 WAF1/CIP 1 via autophagy-PKM2 pathway in human liver cancer stem cells.

### CyclinD1 is required for the oncogenic functions of HULC

**Fig. 7:**
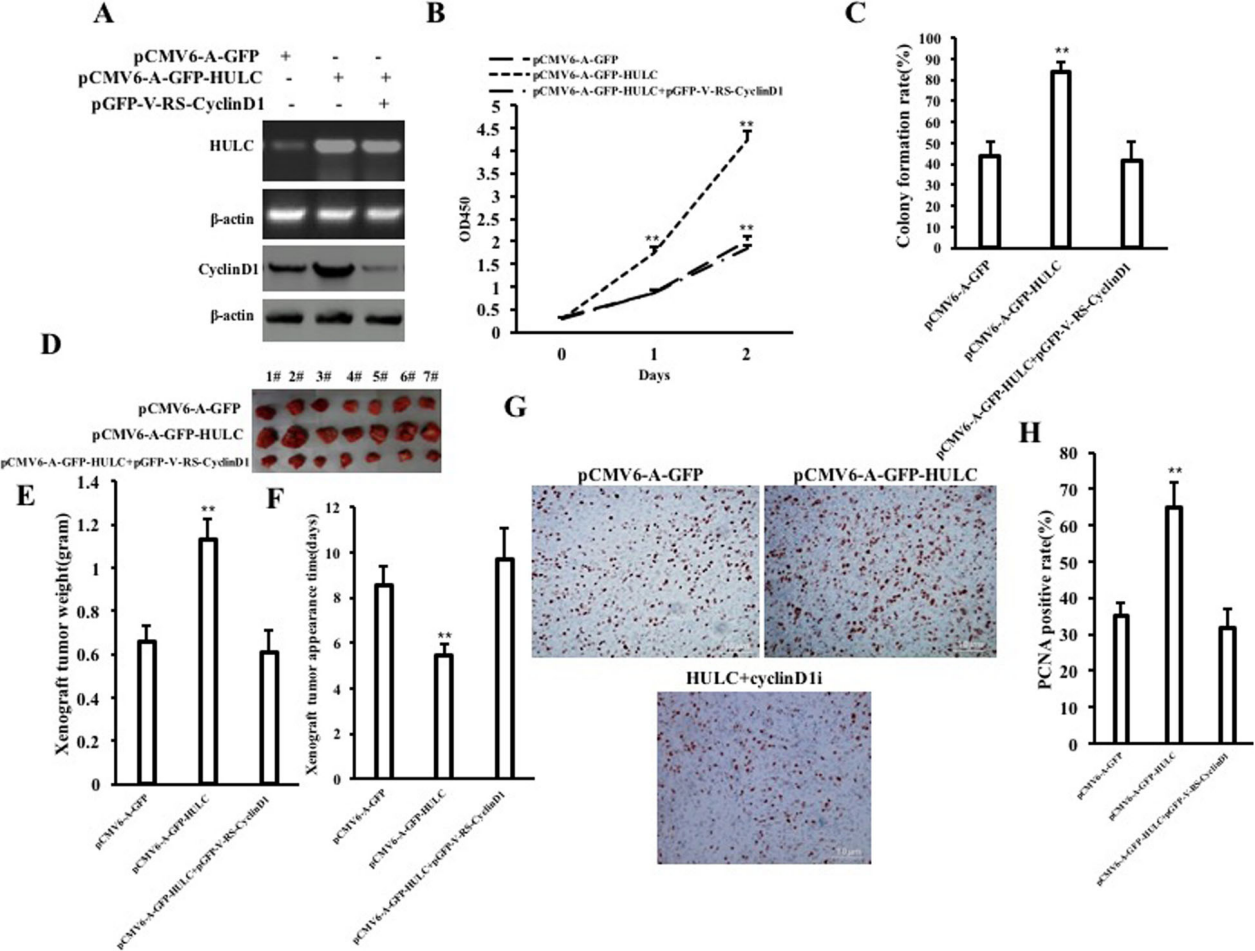
CyclinD1 is required for HULC oncogenic action. **a** RT-PCR for HULC and Western blotting with anti-CyclinD1. β-actin served as internal control. **b** Cell growth assay using CCK8. **c** Colony formation assay. **d** The picture of xenografted tumors from mouse. **e** The wet weight of xenografted tumors from mouse. **f** The appearance time of xenograft tumor. **g** PCNA staining (DAB staining, original magnification× 100). **h** PCNA positive rate analysis

To validate whether CyclinD1 is required for the action of HULC in liver cancer stem cells, we performed the rescued-test. As shown in Fig. 7a, HULC expression was significantly increased in the pCMV6-A-GFP-HULC group and the pCMV6-A-GFP-HULC plus pGFP-V-RS-CyclinD1 group compared with the pCMV6-A-GFP group respectively, and CyclinD1 was significantly increased in the pCMV6-A-GFP-HULC group and decreased in the pCMV6-A-GFP-HULC plus rLV-Cas9-CyclinD1 group compared with the pCMV6-A-GFP group respectively. Next, as shown in Fig. 7b, although the growth of LCSCs was more rapid in the pCMV6-A-GFP-HULC group than in pCMV6-A-GFP (*P* < 0.01), it was not significantly altered in the pCMV6-A-GFP-HULC plus pGFP-V-RS-CyclinD1 group compared to the pCMV6-A-GFP control group (*P* > 0.05). As shown in Fig. 7c, although the colony formation ability was significantly increased in the pCMV6-A-GFP-HULC group compared to the pCMV6-A-GFP group (43.49 ± 6.78% vs 83.86 ± 4.28%, *p* = 0.0097138 < 0.01), it was significantly not altered in the pCMV6-A-GFP-HULC plus pGFP-V-RS-CyclinD1 group compared to the pCMV6-A-GFP group (*P* > 0.05) (43.49 ± 6.78% vs 41.49 ± 9.07%, *p* = 0.14058 > 0.05). As shown in Fig. 7d, e, although the xenograft tumor weight increased approximately twofold in the pCMV6-A-GFP-HULC group compared to the pCMV6-A-GFP group (0.657 ± 0.069 g versus 1.13 ± 0.093 g, *P* = 0.000017 < 0.01), it was not significantly altered in the pCMV6-A-GFP-HULC plus pGFP-V-RS-CyclinD1 group compared to the pCMV6-A-GFP group (0.657 ± 0.069 g versus 0.609 ± 0.101 g, *P* = 0.14394 > 0.05). Although the appearance time of xenograft was significantly decreased in the pCMV6-A-GFP-HULC group compared to the pCMV6-A-GFP group (8.571 ± 0.787 days versus 5.429 ± 0.535 days, *P* = 0.00000991 < 0.01), it was significantly not altered in the pCMV6-A-GFP-HULC plus pGFP-V-RS-CyclinD1 group compared to the pCMV6-A-GFP group (8.571 ± 0.787 days versus 9.714 ± 1.3801 days, *p* = 0.086154 > 0.05) (Fig. 7f). As shown in Fig. 7g, h, although PCNA-positive rate was significantly higher in the pCMV6-A-GFP-HULC group than in the pCMV6-A-GFP group (35.08 ± 3.45% versus 64.83 ± 7.05%, *p* = 0.000101 < 0.01), it was not significantly altered in the pCMV6-A-GFP-HULC plus pGFP-V-RS-CyclinD1 group compared to the pCMV6-A-GFP group (35.08 ± 3.45% versus 31.704 ± 5.143%, *p* = 0.1095 > 0.05). Collectively, findings suggest that HULC accelerates progression of human liver cancer stem cells dependent on CyclinD1.

## Discussion

**Fig. 8:**
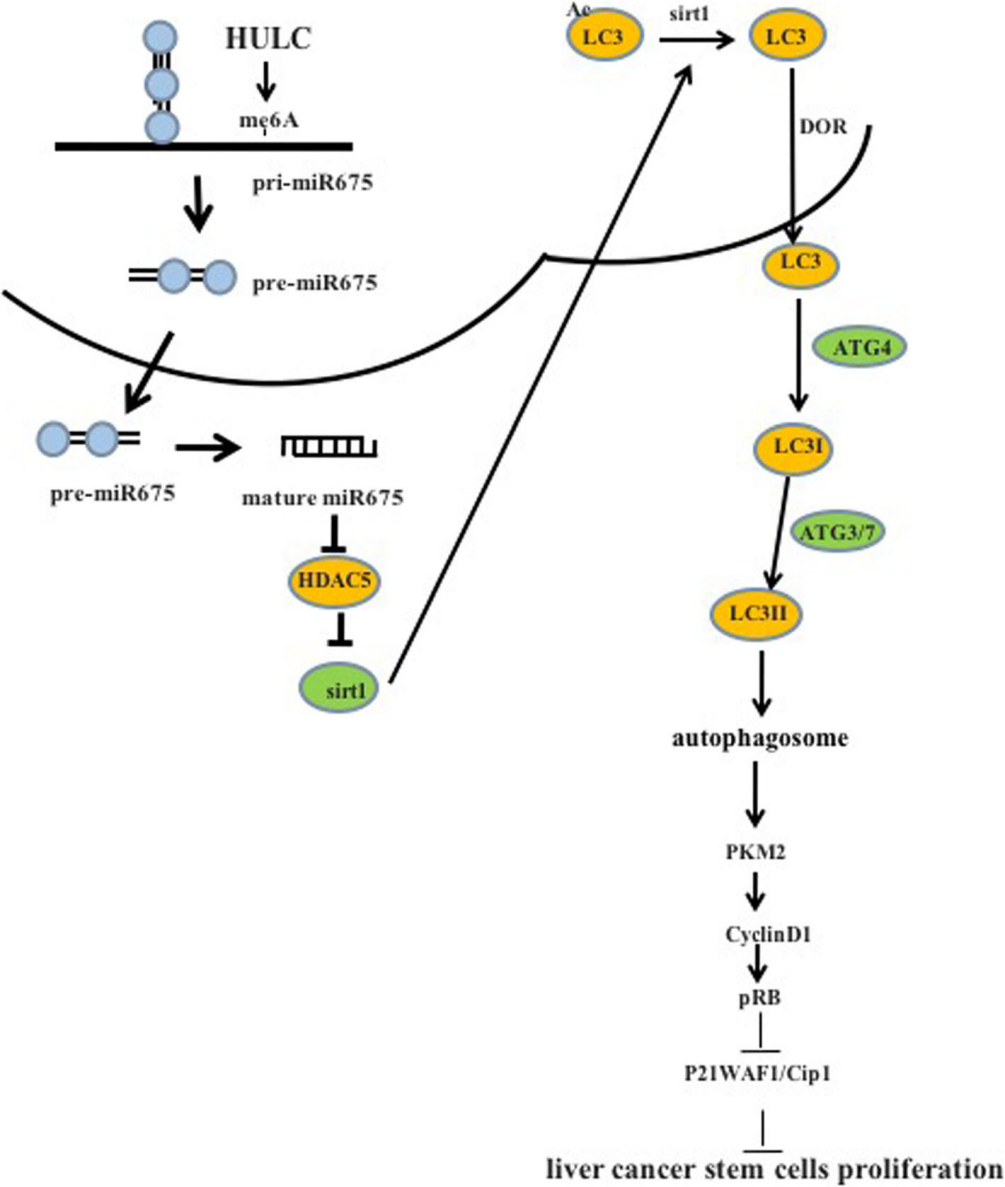
Schematic illustration of the role of HULC in hLCSCs

To date, the functions and regulatory mechanism of long noncoding RNA HULC in liver cancer stem cells have not fully been elucidated. To our knowledge, this paper might be the first to demonstrate that HULC accelerates the growth of human liver cancer stem cells by upregulating CyclinD1 by miR675-PKM2 pathway via autophagy. In this study, we first demonstrate that HULC accelerates growth of liver cancer stem cells in vitro and in vivo*.* Mechanistically, HULC enhances the expression of Sirt1 dependent on miR675 and then induces the cellular autophagy through Sirt1. HULC enhances CyclinD1 and thereby increases pRB and inhibited P21 WAF1/CIP 1 via autophagy-Pyruvate Kinase M2 (PKM2) pathway in human liver cancer stem cells. Ultimately, our results demonstrate that CyclinD1 is required for the oncogenic functions of HULC in human liver cancer stem cells. These observations suggest that HULC accelerates progression of human liver cancer stem cells in vitro *and* in vivo dependent on CyclinD1(Fig. 8).

It is worth mentioning that our findings in this study provide novel evidence for an oncogenic active role of HULC in hLCSCs. This assertion is based on several observations: (a) HULC accelerates growth in vitro of liver cancer stem cells. (b) HULC accelerates growth in vivo of liver cancer stem cells. Several studies indicate that HULC promotes proliferation and migration [34–38]. Our present results are consistent with these reports and provide novel evidence for an active role of HULC in promoting malignant formation and growth of LCSCs. Therefore, we believe that HULC plays a key role in the development of liver cancer.

Importantly, our results suggest that HULC increases the miR675 in human liver cancer stem cells. This assertion is based on several observations: (a) excessive HULC increases and HULC knockdown decreased the binding of METTL3 (a RNA methyltransferase) to pri-miR675. (b) pri-miR675, pre-miR675, and mature miR-R675 were significantly increased in HULC overexpressing hLCSCs. (c) The pre-miR675, pre-miR675, and mature mi-R675 were not significantly altered in the pCMV6-A-GFP-HULC+pGFP-V-RS-METTL3 group. (d) HULC enhances the expression and maturity of miR675 dependent on METTL3 in human liver cancer liver cells. A study showed that miR-675 promoted cancer cell growth [39]. Moreover, miR675 blocks DNA mismatch repair in cancer [40]. Our present results are consistent with these reports and provide novel evidence for an active role of HULC in promoting malignant growth of LCSCs dependent on miR-675. Thus, it suggests that miR675 plays an important role for HULC oncogenic actions.

Evidentially, our findings in this study provide novel evidence that HULC promotes the expression of Sirt1 dependent on miR675. This assertion is based on several observations: (a) miR675 targets HDAC5 mRNA3′-UTR via eight-seed sequence. (b) DHAC5 was significantly reduced in the rLV-miR675 group. (c) The loading of DHAC5 on the Sirt1 promoter region was significantly reduced in the pCMV6-A-GFP-HULC group. (d) The luciferase activity of Sirt1 promoter was significantly increased in the pCMV6-A-GFP-HULC group. (e) The expression of Sirt1 was significantly increased in the pCMV6-A-GFP-HULC group. (f) HULC enhances the expression of Sirt1 dependent on miR675-HDAC5. A study shows that SIRT1 enhances the metabolic flexibility [41]. Moreover, SIRT1 promotes glucose transporting [42] and inhibits apoptosis of cancer cells [43]. Our present results are consistent with these reports. Therefore, miR675-HDAC5-Sirt1 axis regulates the oncogenic functions of HULC. However, it should be explored further.

Notably, our results suggest that HULC increases the autophagy through Sirt1. This evidence is based on results from nine parallel sets of experiments: (a) the interaction between Sirt1 and LC3 was significantly increased in the pCMV6-A-GFP-HULC group. (b) The Ac-LC3 was significantly decreased in the pCMV6-A-GFP-HULC group. (c) The interaction between LC3 and DOR was significantly increased in the pCMV6-A-GFP-HULC group. (d) The interaction between LC3 and ATG4 was significantly increased in the pCMV6-A-GFP-HULC group. (e) The interaction between LC3 and ATG3 was significantly increased in the pCMV6-A-GFP-HULC group. (f) The interaction between ATG3 and ATG7 was significantly increased in the pCMV6-A-GFP-HULC group. (g) The activated LC3II was significantly enhanced in the pCMV6-A-GFP-HULC group. (h) The autophagy was significantly enhanced in the pCMV6-A-GFP-HULC group. (i) HULC increases the autophagy dependent on Sirt1.

Therefore, HULC enhances the expression of Sirt1 dependent on miR675 and therefore increases the autophagy through Sirt1.

Strikingly, HULC enhances CyclinD1 to increase pRB and inhibit P21 WAF1/CIP 1 via autophagy-PKM2 pathway. This evidence is based on results from three parallel sets of experiments: (a) the interaction between LC3II and PKM2 was significantly enhanced in the pCMV6-A-GFP-HULC group. (b) The expression of PI3K and PKM2 was significantly increased in the pCMV6-A-GFP-HULC group. However, the expression of PI3K and PKM2 was not significantly altered in the pCMV6-A-GFP-HULC plus 3-methyladenine (*3-MA*) group. (c) The expression of CyclinD1 was significantly increased in the pCMV6-A-GFP-HULC group. (d) The interaction between CDK4 and CyclinD1 was significantly enhanced in the pCMV6-A-GFP-HULC group. (e) pRB was significantly increased in the pCMV6-A-GFP-HULC group and reduced in the pGFP-V-RS-HULC group. (f) HULC enhances CyclinD1 to increase pRB and inhibit P21 WAF1/CIP1 via autophagy-PKM2 pathway in human liver cancer stem cells. A study shows that autophagy impairs endothelial function [44] and ubiquitination of MAP 1LC3B is associated with autophagy [45]. Interestingly, TLR2 enhances autophagy [46]. Importantly, PKM2 promotes cell survival [47] and regulates STAT3 [48]. In addition, miR-625-5p/PKM2 regulates glycolysis state [49]. Moreover, pRb-E2F pathway induced growth of cancer cells [50]. In particular, microRNA-16-5p modulates Cyclin D1/E1-pRb-E2F1 pathway in cancer cells [51]. Moreover, HPV-16 E7 regulates phospholipase D activity in a pRB-dependent manner [52]. A study shows that lincRNA-p21 acts as a tumor suppressor [53] and SPSB1 destabilizes p21WAF1/Cip1 [54]. Our present results are consistent with these reports and provide novel evidence for oncogenic role of HULC in promoting malignant growth of LCSCs via CyclinD1-pRB-P21WAF1/CIP 1 via autophagy-PKM2 pathway. Therefore, HULC oncogenic action is associated with PKM2, CyclinD1, pRB, P21 WAF1/CIP1, and cellular autophagy in human liver cancer stem cells.

Another significant finding is that CyclinD1 is required for the oncogenic functions of HULC. This evidence is based on results from five parallel sets of experiments: (a) although the growth of LCSCs was more rapid in the pCMV6-A-GFP-HULC group, it was not significantly altered in the pCMV6-A-GFP-HULC plus pGFP-V-RS-CyclinD1 group. (b) Although the colony formation ability of LCSCs was significantly increased in the pCMV6-A-GFP-HULC group, it was not significantly altered in the pCMV6-A-GFP-HULC plus pGFP-V-RS-CyclinD1 group. (c) Although the xenograft tumor weight was increased in the pCMV6-A-GFP-HULC group, it was not significantly altered in the pCMV6-A-GFP-HULC plus pGFP-V-RS-CyclinD1 group. (d) Although the appearance time of xenograft was significantly decreased in the pCMV6-A-GFP-HULC group, it was not significantly altered in the pCMV6-A-GFP-HULC plus pGFP-V-RS-CyclinD1 group. (e) HULC accelerates progression of human liver cancer stem cells dependent on CyclinD1. A study indicated that CyclinD1 polymorphism modified susceptibility oncogene [55]. In particular, miR-760 suppresses cancer growth by targeting cyclinD1 [56]. Furthermore, Cyclin D1 integrates histone methylation [57]. Our present results are consistent with these reports and provide novel evidence for oncogenic role of HULC in promoting malignant growth of LCSCs through CyclinD1.

*In summary*, HULC promotes growth of liver cancer stem cells in vitro and in vivo*.* Mechanistically, HULC enhances the expression of Sirt1 dependent on miR675 and then induces the cellular autophagy through Sirt1. HULC enhances CyclinD1 and thereby increases pRB and inhibited P21 WAF1/CIP 1 via autophagy-PKM2 pathway in human liver cancer stem cells. Ultimately, our results demonstrate that CyclinD1 is required for the oncogenic functions of HULC in human liver cancer stem cells. These observations provide important basic information for finding effective liver cancer therapeutic targets. Therefore, governing HULC expression will be crucial for the identification of novel liver cancer therapeutic strategies. We will further study the exact mechanism of HULC in the development of liver cancer and its clinical application.

## Conclusions

Long noncoding RNA HULC accelerates growth of liver cancer stem cells by enhancing the expression of Sirt1 dependent on miR675 and then inducing the cellular autophagy to increase CyclinD1 and pRB in human liver cancer stem cells. In particular, CyclinD1 is required for the oncogenic functions of HULC in human liver cancer stem cells. These observations provide important basic information for finding effective liver cancer therapeutic targets. Therefore, governing HULC expression will be crucial for the identification of novel liver cancer therapeutic strategies.

## Supplementary information

**Additional file 1: Figure S1.** The isolation and identification of human liver cancer stem cell. A. The transcriptional ability of CD133, CD44, CD24, and Epcam was analyzed by reverse transcription polymerase chain reaction, and β-actin was used as an internal reference gene. B. Western blotting analysis using anti-CD133, anti-CD44, anti-CD24, anti-EpCAM, and β-actin as an internal reference gene. **Figure S2.** A. The assay of sphere formation rate in hLCSCs and non- hLCSCs**.** B. tumorigenesis test in vivo in hLCSCs and non- hLCSCs. **Figure S3. A.** Northern blotting with Biotin-miR675 probe in pCMV6-A-GFP group, pCMV6-A-GFP-HULC group and pCMV6-A-GFP-HULC+pGFP-V-RS-METTL3 group. U6 served as the internal control. B. Real-time RT-PCR with miR675 primers in pCMV6-A-GFP group, pCMV6-A-GFP-HULC group and pCMV6-A-GFP-HULC+pGFP-V-RS-METTL3 group. U6 served as an internal control. **Figure S4.** The analysis of HDAC5 3’UTR (mutant) luciferase reporter activity in rLV-miR675 group and rLV control group.

## Data Availability

Not applicable.

## Abbreviations

HULC: Highly upregulated in liver cancer
RIP: RNA immunoprecipitation
CHIP: Chromatin immunoprecipitation
3′-UTR: 3′ untranslational region
LCSC: Liver cancer stem cell
IGF2BP1: IGF2 mRNA-binding protein 1
SIRT1: Sirtuin 1
*BMP*: Bone morphogenetic protein
HSPC: Hematopoietic stem and progenitor cell
EEF1E1: Eukaryotic translation elongation factor 1 epsilon-1
BNIP3: BCL2 Interacting Protein 3
PKM2: Pyruvate Kinase M2
MALAT1: Metastasis Associated Lung Adenocarcinoma Transcript 1
KDM4B: Lysine-specific demethylase 4B
GLUT1: Glucose transporter protein1
HPV-16: Human papillomavirus 16
HDAC5: Histone deacetylase 5
*3-MA*: 3-Methyladenine
