# Supplementary material for: Long noncoding RNA HULC accelerates the growth of human liver cancer stem cells by upregulating CyclinD1 through miR675-PKM2 pathway via autophagy
Source: Stem Cell Res Ther. 2020 Jan 3;11:8. doi: 10.1186/s13287-019-1528-y (PMC6942366; doi:10.1186/s13287-019-1528-y)
Supplement: Supplementary file 1 — Additional file 1: Figure S1. The isolation and identification of human liver cancer stem cell. A. The transcriptional ability of CD133, CD44, CD24, and Epcam was analyzed by reverse transcription polymerase chain reaction, and β-actin was used as an internal reference gene. B. Western blotting analysis using anti-CD133, anti-CD44, anti-CD24, anti-EpCAM, and β-actin as an internal reference gene. Figure S2. A. The assay of sphere formation rate in hLCSCs and non- hLCSCs. B. tumorigenesis test in vivo in hLCSCs and non- hLCSCs. Figure S3. A. Northern blotting with Biotin-miR675 probe in pCMV6-A-GFP group, pCMV6-A-GFP-HULC group and pCMV6-A-GFP-HULC+pGFP-V-RS-METTL3 group. U6 served as the internal control. B. Real-time RT-PCR with miR675 primers in pCMV6-A-GFP group, pCMV6-A-GFP-HULC group and pCMV6-A-GFP-HULC+pGFP-V-RS-METTL3 group. U6 served as an internal control. Figure S4. The analysis of HDAC5 3’UTR (mutant) luciferase reporter activity in rLV-miR675 group and rLV control group. [file 13287_2019_1528_MOESM1_ESM.docx]

**Supplemental Materials and Methods**

**Human liver cancer stem cell (hLCSC) line sorting** The hLCSCs were isolated from human liver cancer line Huh7 using CD133/CD44/CD24/EpCAM MicroBead Kits(MACS® Technology, Miltenyi Biotech Inc,Boston,USA). and  MACS® Technology operation according to the manufacturer. In brief, centrifuge cell suspension at 300×g for 10 minutes and resuspend human liver cancer cell line Huh7 cell pellet in 300 μl of buffer per 10^8^ total cells after aspirating supernatant completely. Add 100 μL of FcR Blocking Reagent per 10^8^ total cells and 100 μL of CD133/CD44/CD24/EpCAM MicroBeads per 10^8^ total cells. Mix well and incubate for 30 minutes in the refrigerator(2−8 °C).Wash cells by adding 1−2 ml of buffer per 10^8^ cells and centrifuge at 300×g for 10 minutes. Resuspend up to 10^8^ cells in 500 μL of buffer. Choose an appropriate MACS Column and MACS Separator according to the number of total cells and the number of CD133+/CD44+/CD24+/EpCAM+ cells. Human liver cancer stem cell line (hLCSC) was maintained in Dulbecco’s modified Eagle medium(Gibco BRL Life Technologies) or Minimum Essential Medium(MEM) (Gibco BRL Life Technologies) supplemented with 10% heat-inactivated fetal bovine serum (Gibco) in a humidified atmosphere of 5% CO_2_ incubator at 37ºC.

**Cell Transfection** One day before transfection, plate 0.5-2 x 10^5^cells in 500 μl of growth medium without antibiotics so that cells will be 70-90% confluent at the time of transfection.  For each transfection sample, prepare complexes as follows: a. Dilute DNA in 50 μl of Opti-MEM I Reduced Serum Medium without serum. Mix gently. b. Mix Lipofectamine 2000 gently before use, then dilute the appropriate amount in 50 μl of Opti-MEM I Medium. Incubate for 5 minutes at room temperature. Note: Proceed to Step c within 25 minutes. c. After the 5 minute incubation, combine the diluted DNA with diluted Lipofectamine 2000 (total volume = 100 μl). Mix gently and incubate for 20 minutes at room temperature.Add the 100 μl of complexes to each well containing cells and medium. Mix gently by rocking the plate back and forth.Incubate cells at 37°C in a CO_2_ incubator for 18-48 hours prior to testing for transgene expression. Medium may be changed after 4-6 hours.For stable cell lines: Passage cells at a 1:10 (or higher dilution) into fresh growth medium 24 hours after transfection. Add selective medium the following day.

### Western blotting The cells were washed three times with ice-cold phosphate-buffered saline (PBS) and lysed in a lysis buffer. Cells lysates were centrifuged at 12,000g for 20 minutes at 4°C after sonication on ice , and supernatants were separated. Samples were separated on a 10% sodium dodecyl sulfate-polyacrylamide gel electrophoresis (SDS-PAGE) and transferred onto a nitrocellulose membranes, and then blocked in 10% dry milk-TBST (20mM Tris-HCl [PH 7.6], 127mM NaCl, 0.1% Tween 20) for two hours at 37°C. Following three washes in Tris-HCl pH 7.5 with 0.1% Tween 20, the blots were incubated with primary antibody overnight at 4°C. Following three washes, membranes were then incubated with secondary antibody overnight at 4°C. Signals were visualized by enhanced chemiluminescence plus kit (GE Healthcare，Jacksonville, FL, US) according to protocol.

**Co-immunoprecipitation(IP)** Cells were lysed in 1 mL whole-cell extract buffer A(50mM Tris-HCl with a pH of 7.6, 150mMNaCl, 1%NP-40, 0.1mMEDTA,1.0mM DTT,0.2mMPMSF, 0.1mM Pepstatine,0.1mM Leupeptine,and 0.1mM Aproine). 500μL cell lysates were used for immunoprecipitation. Briefly, cell lysates were precleared with 30μL protein G/A-plus agarose beads (Santa Cruz Biotechnology,Inc.,CA, USA) for 1 hour at 4°C and the supernatant was obtained after centrifugation (5000rpm) at 4°C. Precleared supernatant were incubated with 2 µg antibody or normal mouse/rabbit IgG under rotation for 4 hours at 4°C.Then the immunoprecipitates were incubated with 30μL protein G/A-plus agarose beads under rotation overnight at 4°Cand centrifuged at 5000rpm for 5 min at 4°C. The precipitates were washed with beads solution (50 mMTris-HCl with a pH of 7.6,150MmNaCl,0.1% NP-40, and 1mM EDTA) for five times and each time lasted 10 min. Then, the precipitateswere resuspended in 60µL 2×SDS-PAGE sample loading buffer and incubated for 10 min at 100°C. Western blotting was then performed with related antibodies as indicated.

**Chromatin immunoprecipitation (CHIP) assay** Cells were cross-linked with 1% (v/v) formaldehyde (Sigma) for 10 min at room temperature and the crosslinking reaction was stopped after 5 min by addition of 125 mm glycine. Crossed-linked cells were washed with phosphate-buffered saline, resuspended in lysis buffer, and sonicated for 10 min in a SONICS VibraCell to generate DNA fragments with an average size of about 500 bp. Chromatin extracts were diluted 5-fold with dilution buffer, precleared with Protein-A/G-Sepharose beads, and immunoprecipitated with specific antibodies on Protein-A/G-Sepharose beads. After washing, elution and de-cross-linking, the ChIP DNA was detected by PCR.

**Supplemental Figure**

**
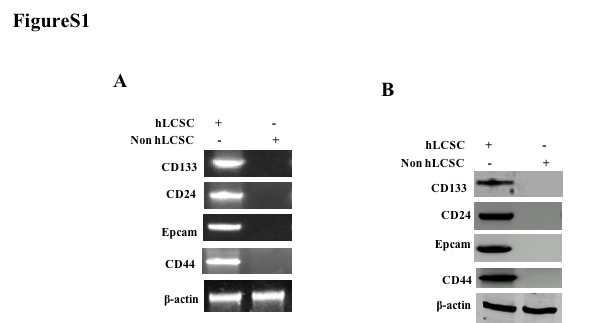
**

**
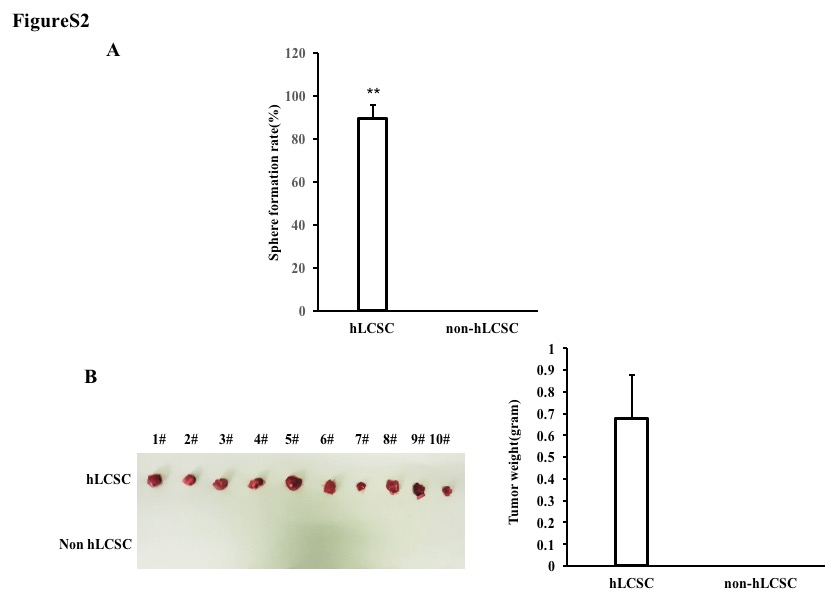
**

**
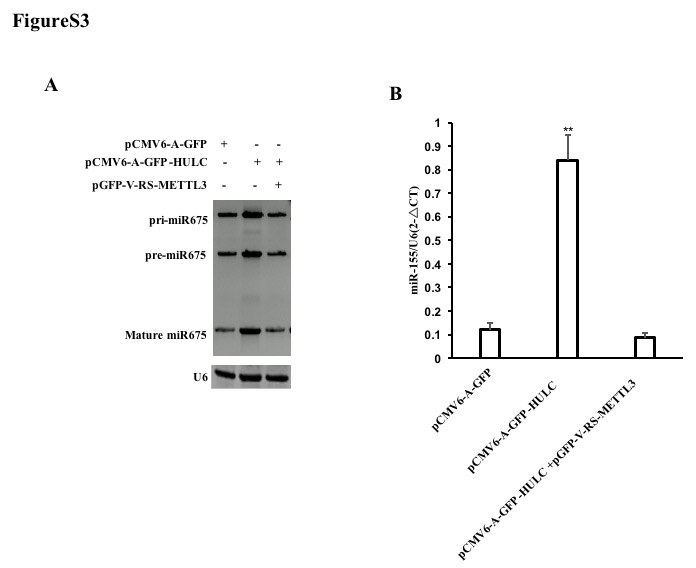
**

**
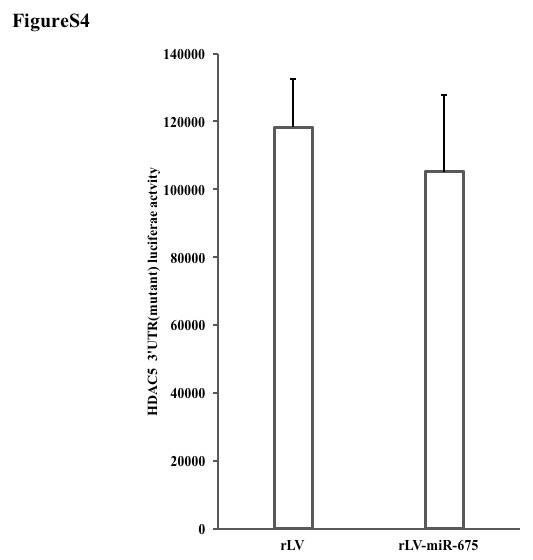
**

**Supplemental Legends**

**Figure S1** The isolation and identification of human liver cancer stem cell. **A.** The transcriptional ability of CD133, CD44, CD24, and Epcam was analyzed by reverse transcription polymerase chain reaction, and β-actin was used as an internal reference gene. B. Western blotting analysis using anti-CD133, anti-CD44, anti-CD24, anti-EpCAM, and β-actin as an internal reference gene.

**Figure S2** A. The assay of sphere formation rate in hLCSCs and non- hLCSCs**.** B. tumorigenesis test *in vivo* in hLCSCs and non- hLCSCs.

**Figure S3 A.** Northern blotting with Biotin-miR675 probe in pCMV6-A-GFP group , pCMV6-A-GFP-HULC group and pCMV6-A-GFP-HULC+pGFP-V-RS-METTL3 group. U6 served as the internal control. **B.** Real-time RT-PCR with miR675 primers in pCMV6-A-GFP group , pCMV6-A-GFP-HULC group and pCMV6-A-GFP-HULC+pGFP-V-RS-METTL3 group. U6 served as an internal control.

**Figure S4** The analysis of HDAC5 3’UTR (mutant) luciferase reporter activity in rLV-miR675 group and rLV control group.
